# Gas Hydrate Combustion in Five Method of Combustion Organization

**DOI:** 10.3390/e22070710

**Published:** 2020-06-27

**Authors:** Sergey Y. Misyura, Andrey Yu. Manakov, Galina S. Nyashina, Olga S. Gaidukova, Vladimir S. Morozov, Sergey S. Skiba

**Affiliations:** 1 Institute of Thermophysics Siberian Branch, Russian Academy of Sciences, 1 Lavrentyev Ave., Novosibirsk 630090, Russia; morozov.vova.88@mail.ru; 2 Nikolaev Institute of Inorganic Chemistry, Siberian Branch, Russian Academy of Sciences, 3 Lavrentyev Ave., Novosibirsk 630090, Russia; manakov@niic.nsc.ru (A.Y.M.); sergey-s-s@mail.ru (S.S.S.); 3 Research School of High-Energy Physics, National Research Tomsk Polytechnic University, 30 Lenin Ave., Tomsk 634050, Russia; gsn1@tpu.ru (G.S.N.); yashutina1993@mail.ru (O.S.G.)

**Keywords:** burning of gas hydrate, dissociation, harmful emissions during combustion

## Abstract

Experiments on the dissociation of a mixed gas hydrate in various combustion methods are performed. The simultaneous influence of two determining parameters (the powder layer thickness and the external air velocity) on the efficiency of dissociation is studied. It has been shown that for the mixed hydrate, the dissociation rate under induction heating is 10–15 times higher than during the burning of a thick layer of powder, when the combustion is realized above the layer surface. The minimum temperature required for the initiation of combustion for different combustion methods was studied. As the height of the sample layer increases, the rate of dissociation decreases. The emissions of NO_x_ and CO for the composite hydrate are higher than for methane hydrate at the same temperature in a muffle furnace. A comparison of harmful emissions during the combustion of gas hydrates with various types of coal fuels is presented. NO_x_ concentration as a result of the combustion of gas hydrates is tens of times lower than when burning coal fuels. Increasing the temperature in the muffle furnace reduces the concentration of combustion products of gas hydrates.

## 1. Introduction

Today, the explored reserves of methane in natural gas hydrate deposits significantly exceed the reserves of natural gas produced by traditional technologies that are not related to the gas-hydrate state [1,2,3]. An overview of existing gas hydrate production technologies and their prospects was given in [3]. Prospects for the use of natural gas hydrates were discussed in [4]. The combustion of coal-based fuels and industrial waste from petrochemical production causes significant damage to the environment and negatively affects global climate warming. Expensive technologies for cleaning harmful emissions are widely used to solve environmental problems. For example, the cleaning technology based on nanofiber filters is being widely used [5]. To reduce concentrations of harmful gases released during combustion, it is possible to effectively use cleaning technologies using carbon nanotubes [6]. Promising technologies for the carbon dioxide capture and storage are discussed in [7].

Another way to reduce harmful emissions in the combustion of various types of coal is the use of coal water slurry [8]. Despite a slight reduction in the amount of harmful emissions, taking into account the applied cleaning technologies, it is impossible to radically solve environmental problems with the widespread burning of coal feedstock. Thus, it is not quite correct to compare the economic efficiency of the burning of coal feedstock and gas hydrates. It is also necessary to account for the costs of developing gas hydrate combustion technologies. The widespread introduction of the latter will reduce harmful emissions into the atmosphere.

However, for the more successful application of new technologies, it is expedient to solve a number of fundamental problems related to the extraction, storage and transportation, as well as to increase the efficiency of the combustion of natural and artificial raw materials in the form of gas hydrates consisting of combustible gases. Today, much attention is paid to the prospects of storing natural and artificial gas-hydrate raw materials at negative temperatures.

Moreover, when the melting point of ice is approached (0 °C), the cost of the raw material cooling becomes lower. The sought storage temperature corresponds to a temperature range from −20 to –(3–5) °C. When the temperature further increases, the plasticity of ice appears, and the phenomenon of “self-preservation” loses its effectiveness. The phenomenon of “self-preservation” refers to abnormally low dissociation rates due to the formation of a high-strength ice crust on the surface of the granules. This high-strength crust enables storing gas-hydrate raw materials for a very long time with insignificant losses of methane, as well as under conditions when the thermodynamic equilibrium is significantly disturbed [9,10,11,12]. Diagnostics of the internal structures of granules using X-ray imaging shows that the dissociation front moves deep into the granule (to the center of the particle) very unevenly during the gas hydrate dissociations. Inhomogeneous areas of ice and gas hydrate arise [13].

Dissociation kinetics depends not only on external and internal conditions of heat exchange, but also on the internal structure of the gas hydrates. For the correct modeling of gas hydrate dissociation kinetics, it is crucial to take into account many conjugate factors, such as the deviation of the pressure and temperature of the ambient medium from the equilibrium state, the size and shape of the particles, the type of unit cell, as well as the “annealing temperature window” when storing feedstock in a certain range of negative temperatures [2]. The most well-known and common types of elementary cells correspond to the structures: (sI, sII, and sH). Gas hydrates with the unit cell of sI, such as methane hydrate, are the most studied and widely used. Hydrates of compound gases (for example, propane-methane) most often have the sII structure. The structural properties of hydrates, as well as the dissociation kinetics under different initial equilibrium conditions (temperature and pressure), are studied in [14,15,16]. The kinetics of dissociation of composite hydrates at temperatures significantly lower than the melting point of ice is studied in [17]. Textural characteristics change over time during gas hydrate dissociations, and the morphology of structures on the surface of the granule changes [17,18]. The kinetic constants of gas hydrates during their dissociation significantly change during the transition from positive to negative temperatures [19]. With a thick layer of powder, it is also necessary to consider the filtration of gas (formed during dissociation) through the powder layer. In addition, the porosity inside the particles (granules) is also realized during dissociation. Regularities of gas flow through a porous medium are considered in [20]. The influence of diffusion and heat fluxes, as well as the size of granules, on the dissociation rate is given in [21,22].

Since natural gas hydrates are usually located in a porous medium at a great depth, there are obvious scientific and technical difficulties in extracting natural raw materials, which are associated with increasing production efficiency, as well as with reducing costs [23]. To date, various schemes have been used to stimulate production: technology based on electrical heating, the depressurization method, and thermal stimulation to increase gas production [24,25,26,27,28]. Experimental studies on the extraction of methane resting in the hydrate-bearing sediments were given in [29]. Simultaneous injection of compressed air and nitrogen allows for increasing the efficiency of methane production [30]. Features of transport and storage of the methane hydrate in the form of a pellet, as well as possible risks, were discussed in [31,32,33,34,35,36,37].

Most studies on the combustion of the gas hydrate powder layer were made for methane hydrate and in the organization of a laminar air flow on the layer surface. This combustion is characterized by unstable behavior of the flame. Even more unstable combustion is observed when burning a thick layer of gas hydrate in a porous rock, when separate “flames” are formed both inside the layer and on its surface [38]. The local areas of burning appear, and then fade. During combustion above the powder layer, uneven voids (cracks in the powder that are filled with methane) appear inside the layer [39]. As a result of the periodic occurrence of these voids, methane is released over the surface of the layer very unevenly, which leads to uneven and unstable combustion. The behavior of the flame during dissociation of methane hydrate in a thick layer is studied in [40,41,42]. The flame front velocity depends on the initial temperature in the sample layer. The combustion of methane hydrate when methane is mixed with air in a narrow gap is given in [43,44]. Due to the fact that a large amount of steam gets into the burning area from the powder layer (with methane hydrate dissociation), the temperature in the flame decreases by more than 150 °C, in comparison with combustion (air + methane) without water vapor. The regularities of methane hydrate combustion in the form of a pressed sphere of large diameter were given in [45]. The change in the size of the sphere over time is very different from the linear relationship that is usually observed for a spherical drop of liquid fuel. Numerical modeling of dissociation and combustion with the appearance of water vapor flow was considered in [46,47]. During combustion of the gas hydrate sphere, most of the water flows down the sphere and does not fall into the burning area, which increases both the rate of dissociation of the gas-hydrate sphere and the burning efficiency (the maximum flame temperature increases noticeably) [48,49]. The use of various oxidizer feed schemes allows for controlling the rate of dissociation of methane hydrate [50]. Nonstationary combustion of gas hydrates was considered in [51,52].

Analysis of the literature has shown that there are very few experimental and theoretical studies comparing the non-isothermal combustion of methane hydrate and a composite gas hydrate, which consists of two different combustible gases. There are no data on the dissociation of mixed hydrate at different sample layer thicknesses. Data are also missing for the comparison of emissions from combustion of methane hydrate, propane hydrate and coal mixtures. In addition, for environmental calculations, experimental data on the influence of gas temperature on the quantitative composition of emissions during combustion are necessary.

## 2. Various Ways of Organizing the Dissociation and Combustion of Gas Hydrates

**Figure 1:**
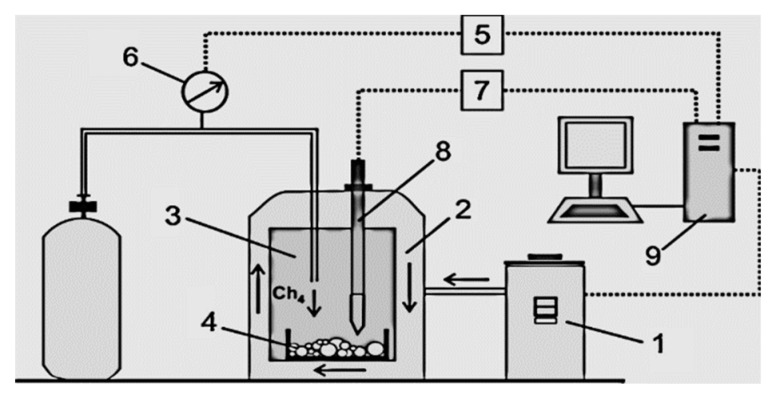
The reactor for synthesizing the powder of simple and mixed gas hydrate: 1—the block of a thermostat to maintain the desired coolant temperature; 2—the reactor coolant; 3—the working part of the reactor for the synthesis of gas hydrate powder; 4—the produced sample (gas hydrate); 5—the gauge pressure inside the reactor; 6—the pressure regulator in the working region of the reactor; 7—the temperature gauge inside the working area of the reactor; 8—thermocouples located directly in the area of gas hydrate synthesis; 9—PC for temperature and pressure control (maintaining the set values of pressure and temperature in the reactor).

Methane hydrate and mixed double hydrate (methane-propane) are synthesized at a high-pressure plant (in the reactor), which is schematically shown in Figure 1. Since the synthesis of gas hydrate, especially methane hydrate, is extremely slow, various methods are used to increase its rate: a magnetic stirrer to intensify the convection in the liquid, nanopowders and various promoters, as well as fine ice to reduce the induction time. For real experiments, fine ice is used to accelerate the synthesis.

Since it is impossible to ensure the growth of powder granules of the same diameter, in order to create particles of similar size, fragmentation and sifting of the particles were used under conditions that excluded gas hydrate dissociation. The sample production process was implemented in several stages (manufacturing, fragmentation, then placing the powder back in the reactor and further synthesis at high pressure in the reactor). The resulting gas hydrate powder consisted of particles with a diameter of 0.2–0.3 mm. It was extremely difficult to control the concentration of methane and propane in the gas-hydrate state, since these concentrations, as well as the filling of elementary cells (the hydrate number) depend on the growth rate and internal mechanisms of kinetics associated with the interaction of molecules in the formation of hydrate. Therefore, in the experiments, mixed methane-propane gas hydrates were used at a single concentration, which was obtained at the set temperature and pressure, i.e., at a certain growth rate. In addition, the article did not set a goal to study the role of the concentration of different gases on the growth kinetics of gas hydrate. Using chromatographic analysis, it has been found that the gas hydrate state has the following ratio of the volume concentration of gases (propane to methane): 60% to 40%. Thus, the amount of propane in the unit cell exceeds the amount of methane. The analysis of the structure of elementary cells has shown that the structure of sI corresponds to the methane hydrate, and for the mixed hydrate, the formula has the form: 16D_1_·8H·136H_2_O (sII structure).

**Figure 2:**
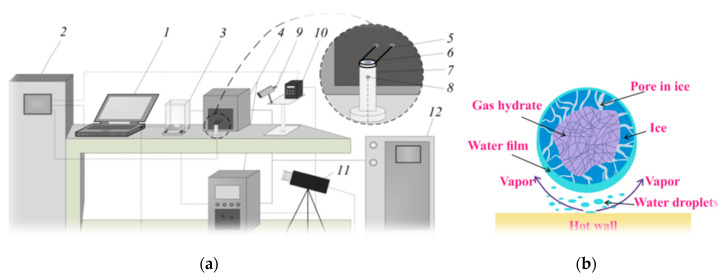
(**a**) Organization of gas hydrate burning using the conductive heating: 1—PC; 2—the multi-channel recorder (for recording data on temperature and video image); 3—the digital scales; 4—the induction heater; 5—the copper coil of the inductor; 6—the gas hydrate; 7—the metal cylinder; 8—the thermoelectric converter; 9—the infrared pyrometer; 10—the temperature controller, 11—the video camera for registering the combustion; 12—the water cooling device; (**b**) a multi-layer scheme for burning gas hydrate granules over the surface of a hot wall at a wall temperature higher than the Leidenfrost temperature.

The method of organizing combustion with the conductive method of heating is shown in Figure 2a. On the upper surface of the metal cylinder, there was a single layer of powder, which consisted of granules of 0.5–1 mm in size.

Since the temperature of the cylinder wall exceeded the temperature of Leidenfrost, the granule was suspended over the wall. During granule combustion a crust of ice forms, with a thin film of water flowing down and forming water vapor. A micron layer of vapor-gas mixture is formed between the granule and the wall and keeps the particle suspended. The high wall temperature is realized by the induction heater (the current frequency of 30–100 kHz, power of 15 kVA, and the maximum current of 22.5 A). The metal cylinder had a radius of 12 mm. The cylinder heating was implemented due to Foucault currents. The error of the thermoelectric converter (in the temperature range of 273–1373 K) did not exceed 3.5 °C. To measure the surface temperature of both the metal cylinder (before placing the gas hydrate) and the powder surface, two methods were used: the thermal imager and the infrared pyrometer with the following parameters: the temperature range within 473 and 1773 K, the error of up to 1 °C, the spectral range within 8 and 14 microns, and the emissivity range within 0.01 and 1.0. The high-speed video camera (11, Phantom-v4, Vision Research) was used to register images of flames. The main technical parameters of video camera are as follows: the maximum resolution of 1280 × 800 pixels; 4200 frames per second; and the sensor pixel size of 20 microns. Software (Tema Automotive, Image Systems AB) was used to process the received video information.

**Figure 3:**
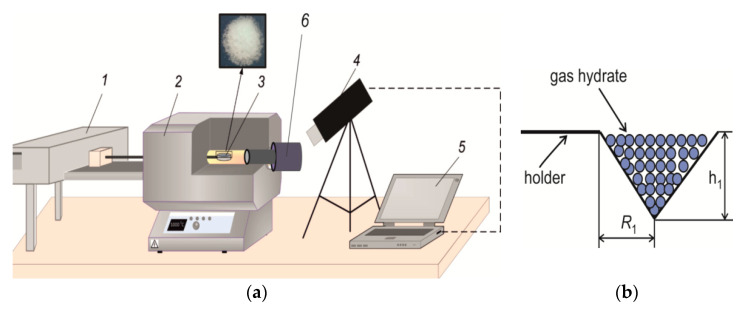
(**a**) Organization of gas hydrate combustion in a muffle furnace: 1—coordinate device for moving the grid with gas hydrate; 2—muffle furnace body; 3—gas hydrate placed in the grid in Figure 4b; 4—the high-speed camera for recording the combustion process through the viewing window; 5—PC; 6—the gas chromatograph for measuring the reaction products during the combustion of gas hydrate; (**b**) geometric parameters of the grid into which the gas hydrate powder was poured.

To change the initial air temperature (over a wide temperature range), it was convenient to use the muffle furnace shown in Figure 3a. With the muffle furnace, experiments were conducted to study the influence of the gas temperature on the concentration of gases released during combustion. The internal diameter of the muffle furnace (Nabertherm R 50/250/13) was 40 mm, and the length of the working area was 450 mm. The furnace was characterized by a fairly uniform temperature field in both the transverse and longitudinal directions (the temperature change in these directions did not exceed 4–5 °C). Before starting the experiment, the powder was weighed on scales and then poured into a grid that had the shape of a cone (Figure 3b). Prior to the experiment, the given temperature was set and maintained in the furnace. The grid with the filled powder was attached to the holder and moved to the working part of the muffle furnace using an automatic movement system. The sample of gas hydrate was located in the center of the furnace. To monitor the combustion process, there was a glass window in the furnace. Using a video camera, the start and stop times of combustion were detected; based on the resulting image of the powder, the residual volume of the sample in the grid was determined.

Measurements of reaction products during combustion of gas hydrates, including methane hydrate and mixed hydrate (methane-propane), were performed using a gas analyzer (Testo 340), whose measuring sensor was inserted into the working volume of the muffle furnace. Measurement of gas composition was realized at different initial temperatures in the furnace (temperature up to the start of combustion of the gas hydrate). The experiments also used a method for organizing combustion due to a hot metal particle. The metal particle (metal cylinder) was first heated in a muffle furnace (Figure 3a) to a predetermined temperature, and then placed over the surface of the gas hydrate powder using a holder. A temperature field formed around the hot particle, which caused the gas hydrate to ignite. Before the experiment, the mass of the gas hydrate was determined by weighing. Next, the powder was placed into a tank (metal bowl). After the experiment, the mass of the remaining powder was also weighed. The temperature of the hot particle varied in the range of 550–1200 °C. The experiments were aimed at determining the minimum temperature at which spontaneous combustion of the gas hydrate begins. The influence of the particle size (metal cylinder), whose diameter varied within 5 and 20 mm, was also studied. Changing the particle size in the specified diameter range had little effect on the minimum temperature that led to combustion.

**Figure 4:**
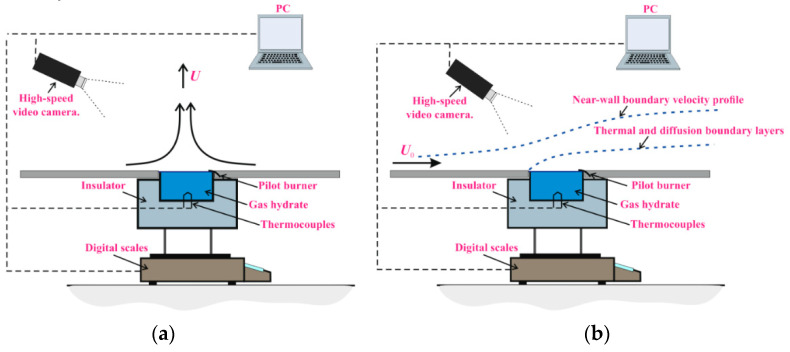
Two different schemes for organizing air motion during gas hydrate combustion: (**a**) absence of external air flow; (**b**) laminar motion of external air with velocity *U*_0_.

Figure 4 shows two schemes for organizing air flow over the gas hydrate powder layer. (a) Free convection of gas over the powder, which is realized due to buoyancy forces. In the combustion of gas hydrate, there are large temperature gradients over the powder in both the longitudinal and transverse direction, which leads to the movement of a mixture of gases (methane-propane-water vapor). (b) There is an interaction of two convections (forced air flow *U*_0_ and free convection due to buoyancy). As a result, the shape of the boundary layers and the shape of the flame differ significantly for the (a) and (b) variants.

In order to reduce the heat flux through the bottom and side walls, the gas hydrate was placed in a rectangular tank with thermoinsulated walls (the wall thickness was 10 mm). After moving the powder from the Dewar vessel, the sample temperature increased due to the presence of heat flux from the ambient medium. The temperature of the ambient air, prior to combustion, was equal to 21–22 °C. Dissociation of gas hydrates and release of methane and propane were realized when the temperature of the powder exceeded the equilibrium temperature at atmospheric pressure. All experiments were performed at an external pressure of 1 bar. When a certain concentration of methane and propane was reached, combustion began in the mixing layer. Combustion leads to an increase in the temperature difference (Δ*T*) between the flame and the powder surface up to 1300–1400 °C (before the start of combustion, the temperature difference between the gas and the surface of the powder layer is 30–40 °C). The increase in heat flux (*q* = α(Δ*T*), α is the gas heat transfer coefficient) leads to an increase in the rate of dissociation of gas hydrates. Thus, the kinetics of combustion and the dissociation rate are interrelated. When the gas hydrate breaks down, ice and gas are formed. Areas of increased sample temperature (60–80 °C) may occur near the layer surface, which leads to a flow of water vapor into the combustion area. The separation and removal of gas during dissociation leads to a decrease in the mass of the powder (∆*M*) over time (*t*). During the entire experiment, the change in the mass of the sample was measured using the gravimetric method (the entire working area with the powder was placed on a Vibra AJH 4200 CE balance). The dissociation rate (*J*) of methane hydrate and mixed hydrate (methane-propane) was determined experimentally as *J* = ∆*M*/∆*t*. The maximum relative error in determining *J* was 6–8%. Inside the powder (in the middle of the layer height and in the center of the working area) there was a thermocouple for measuring the temperature inside the layer. Taking into account the error in the position of the thermocouple, as well as the measurement error due to a loose contact of the sample, the error in measuring the temperature of the layer corresponded to 0.5–1 °C. The thermal field of the layer surface (*T_s_*) (before the beginning of combustion) was measured using a NEC San Instruments thermal imager with a relative measurement error of ±1°C. Before the experiment, the thermal imager was calibrated. When organizing the laminar air flow (Figure 4b), the air velocity *U*_0_ was constant throughout the experiment. During combustion, three characteristic boundary layers were realized above the layer surface (Figure 4b): dynamic (high-speed) and thermal and diffusive wall boundary layers. Moreover, the heat and diffusion layer are located inside the dynamic layer due to the presence of a dynamic background.

## 3. Experimental Measurements and Analysis of Experimental Data

The rate of growth and dissociation of gas hydrate is proportional to the degree of deviation of temperature and pressure from the equilibrium curve. In this case, the reaction rate depends on both the internal kinetics (pre-exponential multiplier) and the activation energy (*E_a_*). In turn, *E_a_* also depends on the internal structure of the gas hydrate (the structure of the unit cell and the nature of the interaction of the gas molecule with water molecules). The above factors are described qualitatively and quantitatively satisfactorily by the phenomenological equations (Equations (1) and (2)) [2,53],

(1) $$-\frac{\Delta m_{i}}{\Delta t}=k_{0}\exp\left(-\frac{E_{a}}{RT}\right)\left(p_{eq}-p\right)s_{i}$$

(2) $$-\frac{\Delta M}{\Delta t}=k_{0}\exp\left(-\frac{E_{a}}{RT}\right)\left(p_{eq}-p\right)S,\;\Delta M=\sum_{i}\Delta m_{i},S\;=\;\sum_{i}s_{i}$$

where *m_i_* is the mass of an individual granule (particle) in the powder, *k*_0_ is the pre-exponential multiplier, which is often expressed through an internal kinetic constant that does not depend on temperature, *p* is the external pressure of gas, *p_eq_* is the equilibrium pressure of gas, which corresponds to the equilibrium curve at a given pressure and temperature, and *s_i_* is the current surface area for a single particle, on which a reaction is implemented for a single granule, and S is the current surface area of reactions for all particles. Taking the reaction surface for all the particles (sum up the current surface for the particles), we obtain the rate of mass change for the entire powder. It is characteristic that at the beginning of dissociation, the reaction area has the maximum value, and when approaching the center of the particle in the final stage of dissociation, the velocity reduces to zero. Thus, the relationship is essentially non-linear. Quasilinearity can be observed only at large distances from the center of the granule, when the derivative of the surface area does not change much along the radius with the time of dissociation. Experimental data show that even at positive sample temperatures, a noticeable non-linearity appears even when the residual mass of the gas hydrate is 30–40% [2]. At negative temperatures and in the area of the annealing temperature window, “self-preservation” strongly affects the nature of the dissociation curve. In this case, the rate of dissociation may fall by several orders of magnitude, compared to the rate of dissociation outside the specified temperature range. It is also important to note that in real conditions, granules (particles) have different sizes, and the temperature is unevenly distributed over the volume of the powder. This leads to the fact that the dissociation rate may differ many times for different particles. Then, the resulting dissociation rate for the entire powder will be the sum of the dissociation rates for all the particles. The activation energy and pre-exponential multiplier at negative powder temperatures will differ significantly from *k*_0_ and *E_a_*, when the dissociation is realized at positive gas hydrate temperatures [19]. When considering different methods of combustion organization, the temperature of the powder will also vary in different ways. In addition, the change in temperature in the layer depends on the layer thickness and the average grain size, as well as on heat flux, which depends on the flame temperature. Theoretically, it is extremely difficult to model all these factors, especially in the presence of negative temperatures. Therefore, experimental studies on the dissociation of gas hydrates in various ways of combustion organization are necessary.

## 4. Dissociation of Gas Hydrate Using Various Schemes of Combustion. Comparison of Combustion Efficiency for Different Methods of Organization of Gas Hydrate Combustion

It has been pointed out above that when the temperature of the hot wall (under the induction heating) is equal to or exceeds a certain critical temperature, which is called the Leidenfrost (*T_L_*) temperature, the particles are in a suspended state (Figure 2b). For stainless steel (the metal cylinder is made of stainless steel), *T_L_* = 270–290 °C. In experiments, the surface temperature of the metal cylinder (on which the powder layer was located) varied in the range of 550–1000 °C, which substantially exceeded the specified *T_L_* value. In this case, the heat flux from the hot wall to the lower surface of the sample granule flows through the vapor-gas layer (Figure 2b) and depends on the thickness of the vapor layer (*δ*). The thickness *δ* has an order of values of approximately 1–10 µm. Despite the fact that the thermal conductivity of the gas is ten times lower than for the gas hydrate, the high temperature difference Δ*Т* = 450–700 °C (between the powder and the solid hot wall) and a small thickness *δ* leads to a higher heat flux and a higher dissociation rate, compared to other gas hydrate combustion methods. A comparison of the rates of dissociation of gas hydrates for different methods will be shown later in table form.

**Figure 5:**
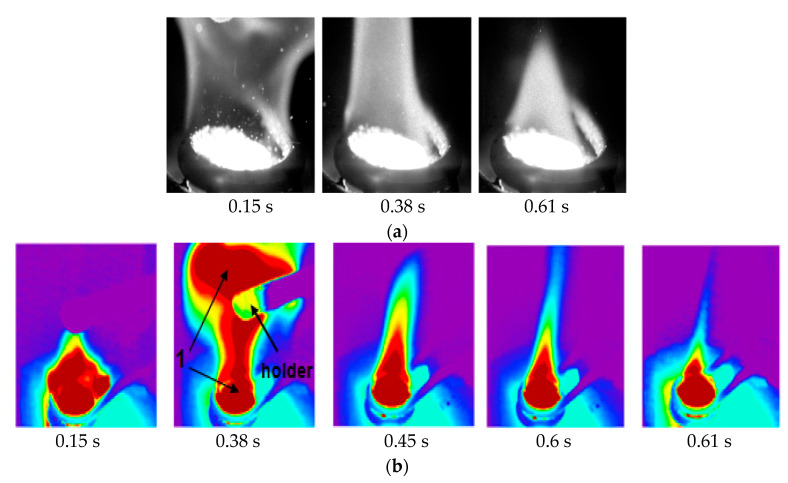
(**a**) Burning of the particles of gas hydrate using induction heating (the initial temperature of the metal cylinder surface of 850 °C); (**b**) the thermal imaging of the flame during methane-propane gas hydrate combustion.

Figure 5a demonstrates photos of gas hydrate powder combustion during induction heating. The maximal flame height is realized at the initial time of combustion. The flame height noticeably changes after 1 s, since the internal part of the granules gets in the area of the annealing temperature window. Since the current mass of the powder was not measured in the experiments, the rate of gas hydrate dissociation was determined under the assumption that the dissociation rate *J* is quasi-constant during combustion. This method of determining *J* gives an error within 10% and can be used for approximate estimates. Then, the dissociation rate can be defined as *J* = ∆*M*/∆*t* = *ρ*·0.11*N*(*V*_i_ -*V_f_*)/*t_c_*, where the mass of the powder *M* is expressed via density *p* and volume *V* of the sample granules in the form of spheres, the subscript *i* corresponds to the initial volume of the powder, and subscript *f* corresponds to the finite volume of the sample at the end of combustion. *N* is the number of gas-hydrate particles on the surface of a hot metal cylinder, and the time *t_c_* corresponds to the duration of the gas hydrate combustion, which was determined using a high-speed camera.

Photos of the flame obtained using a thermal imager are shown in Figure 5b. Since the thermal imager can accurately measure only the surface of a solid or liquid (with appropriate calibration), the photos of the flame itself are provided purely for qualitative analysis of the flame behavior. In the figure, at *t* = 0.15 s, two characteristic combustion regions of methane and propane are clearly visible. One combustion area is located close to the wall (at a distance from the cylinder surface of about 3–7 mm.) The other combustion region (marked with number 1) is located at a great distance from the hot wall (approximately at a distance of 30–50 mm). The gas which is unburnt due to an excess of fuel near the wall burns out at a great distance. The thermal imager also shows that the maximum flame height corresponds to the time *t* = 0.1–0.2 s at *t* = 0.3 s

The height of the flame becomes significantly less. Thus, the maximum dissociation rate of the gas hydrate and the maximum reaction rate during combustion can presumably correspond to the very initial period of combustion. The minimum temperature of the cylinder wall at which combustion started was 655 °C. At a lower temperature, dissociation was realized without combustion.

Let us consider the sample combustion in a muffle furnace (Figure 3). Figure 3b shows the geometric dimensions of the grid with powder, shaped like a cone of revolution (*h*_1_ = 7.5 mm and the radius *R*_1_ = 7.5 mm). As in the case of an induction furnace, the dissociation rate during combustion is determined as *J* = ∆*M*/∆*t* = 0.11·*M_i_*/*t_c_*. If the initial mass of the powder and the duration of combustion are known, then with a quasi-constant *J* over the time of dissociation, it is possible to determine *J* from *M_i_* and *t_c_*. After gas hydrate combustion is completed, the residual mass of the powder is less than 5% of the initial mass of the gas hydrate. Therefore, we can calculate the mass of the released gas by the initial mass and by the given gas concentration in the gas-hydrate state, which corresponds to about 11%. The minimum air temperature in the muffle furnace, at which combustion is realized, corresponds to 570 °C. At a lower temperature in the furnace, dissociation of the gas hydrate occurs without burning.

Let us consider the combustion of gas hydrate at the organization of the beginning of burning from a hot particle (metal cylinder). The surface temperature of the metal cylinder for different experiments varies within 550 and 1200 °C. The combustion the of gas hydrate starts when the temperature reaches 1100 °C and higher. This temperature of combustion is noticeably higher than in other combustion methods under study. The distance *h_c_* between the lower surface of the hot cylinder and the upper surface of the sample layer at which combustion occurred (at *T* = 1100 °C) was approximately 4–5 mm. When the hot particle is further removed from the powder surface, combustion does not occur due to a high temperature gradient near the surface of the particle. The stoichiometric ratio (when methane is burnt above the powder surface) is performed at a distance from the surface of the sample layer of *h_s_* = 1.5–2 mm [41]. At the beginning of combustion from a metal particle, the distance *h_c_* exceeds the distance *h_s_* several times. Thus, combustion beginning depends not only on the place where the stoichiometric ratio is performed, but it is also important to know the minimum temperature below which there is no burning.

**Figure 6:**
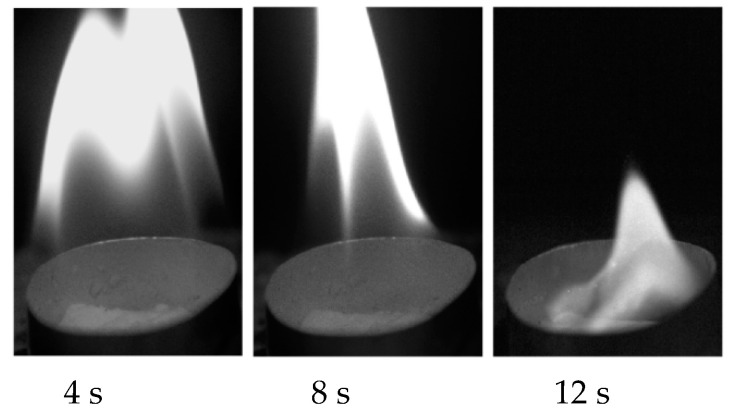
Organization of the beginning of combustion of mixed hydrate powder (methane-propane) from a hot metal cylinder (*T_s_* = 1100 °C).

Pictures of the flame shape during the combustion of mixed hydrate powder (methane-propane) from the hot particle are shown in Figure 6.

Combustion of the powder (from the hot particle) occurs in the absence of external forced air flow. Air movement is realized due to buoyancy (the direction of gas movement corresponds to Figure 4a). In this case, the flame shape and combustion are more stable than in the presence of *U*_0_ (Figure 4b). The external air velocity leads to a change in the flame shape, as well as to a change in the heat exchange conditions between the ambient medium and the powder layer. The heat flux *q* (*q* = αΔ*T*), directed from the gas to the surface of the powder is proportional to the heat transfer coefficient α in the gas phase. The coefficient α increases with the growth of the Reynolds number α ~ (*Re*)^0^.^5^ [54,55]. Then, α ~ (*U_0_*)^0^.^5^. However, when two convections are superimposed (forced external flow and free thermogravitational convection), the dependence of heat transfer and dissociation rate on *U_0_* will be more difficult due to the change in the shape of the flame. In addition, the heat transfer in the powder layer depends on the layer thickness (*q* ~ λ(Δ*T*/*l*), where λ is the thermal conductivity in the sample layer, Δ*T* is the temperature difference between the upper and lower layer surface, and *l* is the layer thickness). The higher the thickness of the powder layer is, the lower the heat flux and dissociation rate *J* (*J* ~ (*q*)^n^) will be, since the average temperature throughout the entire layer volume will be growing more slowly with the growth of the value *l*.

**Figure 7:**
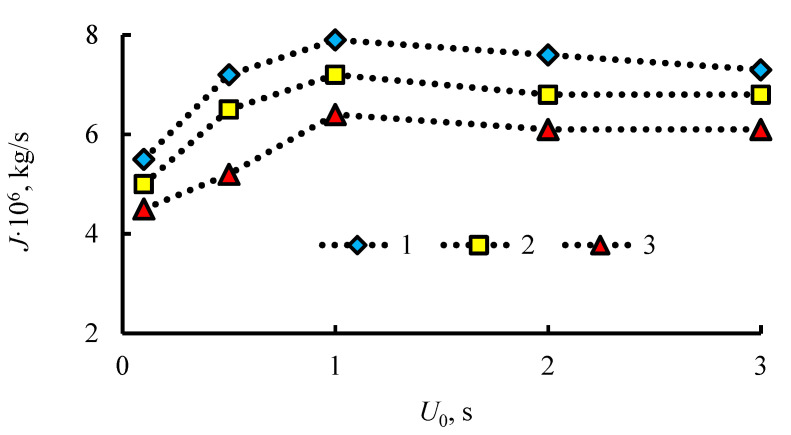
Dependence of the dissociation rate of mixed hydrate (propane-methane) *J* on the velocity *U_0_* and the height of the powder layer *l*: 1 – *l* = 13 mm; 2 – *l* = 18 mm; 3 – *l* = 23 mm.

Figure 7 shows experimental curves for the effect of the forced air flow velocity *U_0_* and the height of the powder layer of mixed hydrate (propane-methane) *l* on the dissociation rate *J*.

As the velocity increases, two characteristic dissociation modes are implemented. For 0 < *U_0_* < 0.8–1.2, the rate of dissociation of the mixed hydrate increases, which corresponds to the above analysis for the intensification of heat exchange and dissociation with an increase in the rate. With a further increase in *U_0_*, the dissociation rate decreases, which is probably due to a change in the shape of the flame. The flame (the combustion region) becomes more strongly pressed against a colder wall. During the combustion of liquid fuel, the maximum temperature is in the vicinity of the liquid surface. The surface of the gas hydrate has a low temperature due to the melting of ice and evaporation of a thin film of water. When combustion comes close to the wall area with a low temperature, then the burning temperature also decreases, which causes a drop in *J*. As can be seen from the experimental graphs, the dissociation rate decreases markedly with an increase in the layer thickness *l*. The reason for this drop is discussed above and is associated with a decrease in the transverse heat flux in the powder layer when *l* increases.

The comparison of experimental data on the dissociation rate (*J*_1_) for mixed gas hydrate (propane-methane) with the use of various combustion options is shown in Table 1. The maximum dissociation rate is achieved by the induction heating of a thin layer of powder, since in this case the layer thickness coincides with the diameter of the particles. In addition, it was indicated above that a micron vapor layer occurs between the wall and the powder. Due to its small height, as well as due to the high temperature gradient in this vapor layer, a high heat flux is realized, leading to a high dissociation rate of the mixed hydrate. The minimum dissociation rate is specific for a thick layer of powder (the powder layer without *U*_0_). Table 2 shows data for the minimum temperature *T*, at which the combustion of the mixed hydrate starts.

The minimum temperature corresponds to the mixed hydrate heating in the muffle furnace (*T_min_* = 635–655 °C). In this case, no additional heating of cold atmospheric air is required. The maximum temperature value (*T_min_* = 1990–1100 °C) corresponds to heating using a hot particle (a hot cylinder that has been preheated in a muffle furnace). The surface temperature of the cylinder during induction heating and the surface temperature of the hot particle are measured by a thermal imager.

## 5. Measuring Gas Concentration during Gas Hydrate Combustion

Combustion of coal-based fuels in modern power plants is accompanied by the release of a large number of environmentally harmful gases. As a result, cleaning gases from harmful combustion products and their disposal require huge financial resources. This paper presents a comparison of concentrations of several types of gases during combustion of (1) mixed methane-propane hydrate, (2) methane gas hydrate, and (3) comparison with coal and water-coal mixtures.

**Figure 8:**
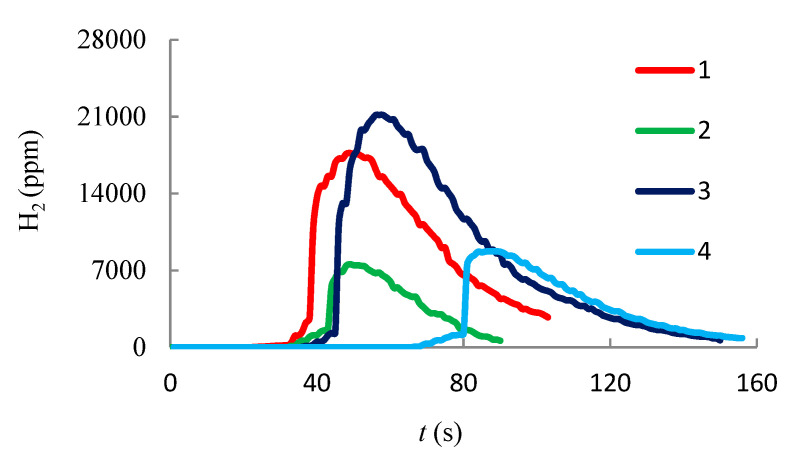
Ppm for H_2_: (1, 2)—during methane hydrate combustion; (3, 4)—during mixed hydrate combustion (methane-propane); (1, 3)—*T* = 600 °C; (2, 4)—*T* = 1000 °C.

**Figure 9:**
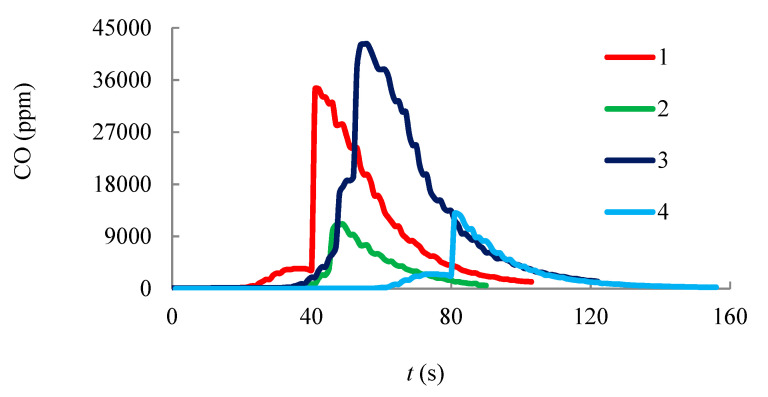
Ppm for CO: (1, 2)—during methane hydrate combustion; (3, 4)—during mixed hydrate combustion (methane-propane); (1, 3)—*T* = 600 °C; (2, 4)—*T* = 1000 °C.

**Figure 10:**
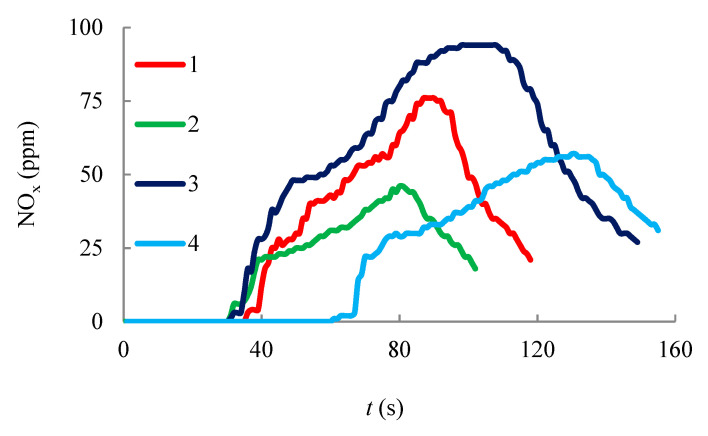
ppm for NO_x_: (1, 2)—during methane hydrate combustion; (3, 4)—during mixed hydrate combustion (methane-propane); (1, 3)—*T* = 600 °C; (2, 4)—*T* = 1000 °C.

Gas sampling for Testo 340 analyzer is performed in a muffle furnace (Figure 3a). The electrochemical sensors determine the concentration of gases: NO_x_, СО and H_2_. The results of chromatographic measurements are shown in Figure 8, Figure 9 and Figure 10. Gas emissions are measured at different temperatures inside the muffle furnace.

It is apparent from the graphs that the amount of NO_x_ and СО is higher for the mixed hydrate than for the methane hydrate, which is probably due to the difference in the structure of the unit cell (higher carbon content in the hydrate cell of the mixed hydrate), as well as a higher concentration of water vapor in the flame. As the initial air temperature in the muffle furnace increases, the amount of measured gases decreases, which indicates a higher reaction rate when burning at a higher temperature of the external air. Under the action of buoyancy, free convection is organized and external air enters the region of combustion. The higher the initial temperature of the outside air is, the less energy is spent on heating it up to the combustion temperature. Thus, to reduce CO and H_2_ emissions, it is expedient not only to increase the temperature in the combustion area by reducing the concentration of steam, but also to increase the temperature of the oxidizer entering the combustion area. Experimental data on measuring NO_x_, CO and H_2_ concentrations during the combustion of methane hydrate and mixed gas hydrate are shown in Table 3 (each value in the table is the average of three repeated experiments).

The greatest decrease in concentration when the temperature increases is observed for CO, which is probably the most sensitive to the temperature in the flame and to the reaction rate during combustion. It is also important to compare the results obtained for gas hydrate with other widely used fuels (coal, the coal mixtures [56], the filter-cake (wet) and the coal-water slurry for coal of 50% and water of 50%). Experimental data on the gas harmful emissions are shown in Table 4, Table 5, Table 6 and Table 7. Experimental data on the harmful emissions of NO_x_ during coal fuel combustion (Table 6) show that the concentration of this gas is tens of times higher than during the burning of the used gas hydrates. At the same time, there are no emissions of SO_x_ during gas hydrate burning. The SO_x_ concentration during the combustion of coal fuel is rather high, which causes significant environmental damage. CO and H_2_ concentrations are slightly higher in the combustion of mixed hydrate than for coal mixtures, which is associated with a high concentration of steam in the combustion region. Removing water from the gas hydrate layer during combustion will reduce the concentration of these emissions, which is the subject of further research.

In the combustion of the sphere of methane hydrate, the spontaneous removal of water is realized, not only leading to increased combustion temperature, but also increasing the dissociation rate of gas hydrate spheres, and changing the rate of elementary reactions in combustion [46,47]. The organization of water drainage is one of the effective ways to reduce the concentration of the reaction products during combustion.

## 6. Conclusions

For the mixed gas hydrate (propane-methane), the dissociation rate has been compared for different combustion methods. The maximum rate of dissociation corresponds to the combustion during the induction heating of a single-layer flow, when the height of the layer is equal to the diameter of the powder granules. The minimum dissociation rate is realized for a thick layer of powder without an external forced air flow. At the first specified option (induction heating), the dissociation rate is 10–15 times higher.

An increase in the thickness of the powder layer leads to a decrease in the rate of dissociation of the mixed gas hydrate. The influence of the external gas velocity on the rate of dissociation of the gas hydrate is nonlinear.

NO_x_ and CO emissions are shown to be higher when burning the mixed hydrate (propane-methane) than when burning the methane hydrate at the same temperature in the muffle furnace. Increasing the temperature in the muffle furnace reduces the concentration of combustion products of gas hydrates.

The experimental data on harmful emissions of gas hydrates and carbon fuels during their combustion have been compared using a gas analyzer. NO_x_ concentration (in the combustion of gas hydrates) is tens of times lower than in the combustion of coal fuels.

## Figures and Tables

**Table 1:** The experimental data on the dissociation rate *J*_1_ = *J*/*S*_1_ (*S*_1_ is the top surface area of the powder layer) of the mixed gas hydrate of propane and methane with the following burning variants: (1) burning of one layer (the layer height equal to the diameter of granules of 0.5–1 mm) during induction heating; (2) combustion in the muffle furnace; (3) initiation of combustion of gas hydrate using the hot cylinder located above the powder surface; (4) combustion of the powder layer without external forced air flow (*U_0_* = 0 m/s, layer height *l* = 18 mm/s); (5) the burning of the powder layer in the presence of a forced external flow of air (*U_0_* = 1.2 m/s, *l* = 18 mm/s).

	Induction Heating	Muffle Furnace	Heating with Hot Cylinder	Powder Layer without *U*_0_	Powder Layer (*U*_0_ = 1.2 m/s)
*J*_1_, kg/(s∙m^2^)	0.03–0.04	0.014–0.016	0.0025–0.0027	0.0024–0.0026	0.0034–0.0035

**Table 2:** Minimum temperature *T_min_*, at which the combustion of mixed hydrate (propane-methane) is realized: (1) induction heating of mixed hydrate; (2) burning of gas hydrate in the muffle furnace; (3) burning with the hot cylinder.

	Induction Heating	Muffle Furnace	Heating with Hot Cylinder
*T_min_*, °C	653–655	568–570	1990–1100

**Table 3:** Measurements of SO_x_, NO_x_, CO and H_2_ concentrations during the combustion of methane hydrate and mixed hydrate (propane-methane) with air temperature changes in the muffle furnace.

*T*, °C	H_2_, % Methane Hydrate	H_2_, % Mixed Hydrate	CO, % Methane Hydrate	CO, % Mixed Hydrate	NO_x_, % Methane Hydrate	NO_x_, % Mixed Hydrate	SO_x_, %
600	0.87	0.97	0.69	0.79	0.003	0.0036	-
700	0.76	0.82	0.63	0.69	0.0026	0.0036	-
800	0.57	0.66	0.50	0.58	0.0023	0.0034	-
900	0.52	0.61	0.37	0.43	0.0018	0.003	-
1000	0.45	0.54	0.29	0.35	0.0015	0.0027	-

**Table 4:** Concentration of H_2_ during the combustion of coal and coal mixture (*T*—temperature in the muffle furnace).

*T*, °C	Coal, %	Filter-Cake (wet), %	Coal-Water Slurry (Coal 50%, Water 50%)
700	0.32	0.35	0.34
800	0.18	0.27	0.3
900	0.13	0.22	0.23

**Table 5:** CO concentration during the combustion of coal and coal mixture (*T*—temperature in the muffle furnace).

*T*, °C	Coal, %	Filter-Cake (wet), %	Coal-Water Slurry (Coal 50%, Water 50%)
700	0.14	0.2	0.19
800	0.12	0.17	0.15
900	0.08	0.99	0.12

**Table 6:** NO_x_ concentration during the combustion of coal and coal slurry (*T*—temperature in the muffle furnace).

*T*, °C	Coal, %	Filter-Cake (Wet), %	Coal-Water Slurry (Coal 50%, Water 50%)
700	0.014	0.008	0.012
800	0.018	0.017	0.015
900	0.032	0.026	0.023

**Table 7:** Concentration of SO_x_ during the combustion of coal and coal mixture (*T*—temperature in the muffle furnace).

*T*, °C	Coal, %	Filter-Cake (Wet), %	Coal-Water Slurry (Coal 50%, Water 50%)
700	0.0065	0.0017	0.001
800	0.0021	0.0055	0.0032
900	0.0027	0.0094	0.007

## References

[1] Sum A.K., Koh C.A., Sloan E.D. (2009). Clathrate Hydrates: From Laboratory Science to Engineering Practice. Ind. Eng. Chem. Res..

[2] Istomin V.A., Yakushev V.S. (1992). Gas hydrates in nature. Mar. Geol..

[3] Cui Y., Lu C., Wu M., Peng Y., Yao Y., Luo W. (2018). Review of exploration and production technology of natural gas hydrate. Adv. Geo Energy Res..

[4] Chong Z.R., Yang S.H.B., Babu P., Linga P., Li X.-S. (2016). Review of natural gas hydrates as an energy resource: Prospects and challenges. Appl. Energy.

[5] Liu Y., Su Y., Guan J., Cao J., Zhang R., He M., Jiang Z. (2018). Asymmetric aerogel membranes with ultra-fast water permeation for separation of oil-in-water emulsion. ACS Appl. Mater. Interfaces.

[6] Mantzalis D., Asproulis N., Drikakis D. (2011). Filtering carbon dioxide through carbon nanotubes. Chem. Phys. Lett..

[7] Leung D.Y., Caramanna G., Maroto-Valer M. (2014). An overview of current status of carbon dioxide capture and storage technologies. Renew. Sustain. Energy Rev..

[8] Dmitrienko M.A., Nyashina G.S., Strizhak P.A. (2017). Environmental indicators of the combustion of prospective coal water slurry containing petrochemicals. J. Hazard. Mater..

[9] Takeya S., Yoneyama A., Ueda K., Mimachi H., Takahashi M., Sano K., Hyodo K., Takeda T., Gotoh Y. (2012). Anomalously Preserved Clathrate Hydrate of Natural Gas in Pellet Form at 253 K. J. Phys. Chem. C.

[10] Kuhs W.F., Genov G., Staykova D.K., Hansen T.C. (2004). Ice perfection and onset of anomalous preservation of gas hydrates. Phys. Chem. Chem. Phys..

[11] Zhang G., Rogers R.E. (2008). Ultra-stability of gas hydrates at 1atm and 268.2K. Chem. Eng. Sci..

[12] Takeya S., Ripmeester J.A. (2010). Anomalous Preservation of CH4Hydrate and its Dependence on the Morphology of Hexagonal Ice. ChemPhysChem.

[13] Takeya S., Yoneyama A., Ueda K., Hyodo K., Takeda T., Mimachi H., Takahashi M., Iwasaki T., Sano K., Yamawaki H. (2011). Nondestructive Imaging of anomalously preserved methane clathrate hydrate by phase contrast X-ray imaging. J. Phys. Chem. C.

[14] Stern L.A., Circone S., Kirby S.H., Durham W.B. (2001). Anomalous Preservation of Pure Methane Hydrate at 1 atm. J. Phys. Chem. B.

[15] Stern L.A., Cirсone S., Kirby S.H., Durham W.B. (2003). Temperature, pressure and compositional effects on anomalous or “self” preservation of gas hydrates. Can. J. Phys..

[16] Shimada W., Takeya S., Kamata Y., Uchida T., Nagao J., Ebinuma T., Narita H. (2005). Texture Change of Ice on Anomalously Preserved Methane Clathrate Hydrate. J. Phys. Chem. B.

[17] Prasad P.S.R., Chari V.D. (2015). Preservation of methane gas in the form of hydrates: Use of mixed hydrates. J. Nat. Gas Sci. Eng..

[18] Falenty A., Kuhs W.F. (2009). “Self-Preservation” of CO2Gas Hydrates—Surface Microstructure and Ice Perfection. J. Phys. Chem. B.

[19] Misyura S., Donskoy I. (2019). Ways to improve the efficiency of carbon dioxide utilization and gas hydrate storage at low temperatures. J. CO2 Util..

[20] Singh H., Myong R. (2018). Critical Review of Fluid Flow Physics at Micro-to Nano-scale Porous Media Applications in the Energy Sector. Adv. Mater. Sci. Eng..

[21] Misyura S. (2013). Effect of heat transfer on the kinetics of methane hydrate dissociation. Chem. Phys. Lett..

[22] Misyura S., Donskoy I. (2016). Dissociation of natural and artificial gas hydrate. Chem. Eng. Sci..

[23] Wang Y., Feng J.-C., Li X.-S., Zhan L., Li X.-Y. (2018). Pilot-scale experimental evaluation of gas recovery from methane hydrate using cycling-depressurization scheme. Energy.

[24] Li B., Xu T., Zhang G., Guo W., Liu H., Wang Q., Qu L., Sun Y. (2018). An experimental study on gas production from fracture-filled hydrate by CO_2_ and CO_2_/N_2_ replacement. Energy Convers Manag..

[25] Tupsakhare S.S., Castaldi M.J. (2019). Efficiency enhancements in methane recovery from natural gas hydrates using injection of CO2/N2 gas mixture simulating in-situ combustion. Appl. Energy.

[26] Li B., Liu S.D., Liang Y.P., Liu H. (2018). The use of electrical heating for the enhancement of gas recovery from methane hydrate in porous media. Appl. Energy.

[27] Rossi F., Gambelli A.M., Sharma D.K., Castellani B., Nicolini A., Castaldi M.J. (2019). Experiments on methane hydrates formation in seabed deposits and gas recovery adopting carbon dioxide replacement strategies. Appl. Therm. Eng..

[28] Wang Y., Feng J.-C., Li X.-S., Zhang Y. (2018). Influence of well pattern on gas recovery from methane hydrate reservoir by large scale experimental investigation. Energy.

[29] Okwananke A., Hassanpouryouzband A., Farahani M.V., Yang J., Tohidi B., Chuvilin E., Istomin V., Bukhanov B. (2019). Methane recovery from gas hydrate-bearing sediments: An experimental study on the gas permeation characteristics under varying pressure. J. Pet. Sci. Eng..

[30] Okwananke A., Yang J., Tohidi B., Chuvilin E., Istomin V., Bukhanov B., Cheremisin A. (2018). Enhanced depressurisation for methane recovery from gas hydrate reservoirs by injection of compressed air and nitrogen. J. Chem. Thermodyn..

[31] Rehder G., Eckl R., Elfgen M., Falenty A., Hamann R., Kähler N., Kuhs W.F., Osterkamp H., Windmeier C. (2012). Methane Hydrate Pellet Transport Using the Self-Preservation Effect: A Techno-Economic Analysis. Energies.

[32] Sloan E.D., Koh C.A. (2008). Clathrate Hydrates of Natural Gases.

[33] Takahashi M., Moriya H., Katoh Y., Iwasaki T. Development of natural gas hydrate (NGH) pellet production system by bench scale unit for transportation and storage of NGH pellet. Proceedings of the 6th International Conference on Gas Hydrates.

[34] Kim N.-J., Kim C.B. (2004). Study on gas hydrates for the solid transportation of natural gas. KSME Int. J..

[35] Javanmardi J., Nasrifar K., Najibi S., Moshfeghian M. (2005). Economic evaluation of natural gas hydrate as an alternative for natural gas transportation. Appl. Therm. Eng..

[36] Mimachi H., Takahashi M., Takeya S., Gotoh Y., Yoneyama A., Hyodo K., Takeda T., Murayama T. (2015). Effect of Long-Term Storage and Thermal History on the Gas Content of Natural Gas Hydrate Pellets under Ambient Pressure. Energy Fuels.

[37] Kim K., Kang H., Kim Y. (2015). Risk Assessment for Natural Gas Hydrate Carriers: A Hazard Identification (HAZID) Study. Energies.

[38] Chen X.-R., Li X., Chen Z., Zhang Y., Yan K.-F., Lv Q.-N. (2015). Experimental Investigation into the Combustion Characteristics of Propane Hydrates in Porous Media. Energies.

[39] Misyura S., Misyura S. (2019). Non-stationary combustion of natural and artificial methane hydrate at heterogeneous dissociation. Energy.

[40] Maruyama Y., Yokomori T., Ohmura R., Ueda T. Flame spreading over combustible hydrate in a laminar boundary layer. Proceedings of the 7th International Conference on Gas Hydrate.

[41] Maruyama Y., Fuse M.J., Yokomori T., Ohmura R., Watanabe S., Iwasaki T., Iwabuchi W., Ueda T. (2013). Experimental investigation of flame spreading over pure methane hydrate in a laminar boundary layer. Proc. Combust. Inst..

[42] Nakamura Y., Katsuki R., Yokomori T., Ohmura R., Takahashi M., Iwasaki T., Uchida K., Ueda T. (2009). Combustion Characteristics of Methane Hydrate in a Laminar Boundary Layer. Energy Fuels.

[43] Wu F., Padilla R., Dunn-Rankin D., Chen G., Chao Y.-C. (2017). Thermal structure of methane hydrate fueled flames. Proc. Combust. Inst..

[44] Chien Y.-C., Dunn-Rankin D. (2019). Combustion Characteristics of Methane Hydrate Flames. Energies.

[45] Yoshioka T., Yamamoto Y., Yokomori T., Ohmura R., Ueda T. (2015). Experimental study on combustion of a methane hydrate sphere. Exp. Fluids.

[46] Bar-Kohany T., Sirignano W.A. (2016). Transient combustion of a methane-hydrate sphere. Combust. Flame.

[47] Dagan Y., Bar-Kohany T. (2018). Flame propagation through three-phase methane-hydrate particles. Combust. Flame.

[48] Cui G., Wang S., Dong Z., Xing X., Shan T., Li Z. (2020). Effects of the diameter and the initial center temperature on the combustion characteristics of methane hydrate spheres. Appl. Energy.

[49] Cui G., Dong Z., Wang S., Xing X., Shan T., Li Z. (2020). Effect of the water on the flame characteristics of methane hydrate combustion. Appl. Energy.

[50] Misyura S. (2016). Efficiency of methane hydrate combustion for different types of oxidizer flow. Energy.

[51] Misyura S.Y., Nakoryakov V.E. (2013). Nonstationary Combustion of Methane with Gas Hydrate Dissociation. Energy Fuels.

[52] Misyura S., Donskoy I. (2020). Dissociation kinetics of methane hydrate and CO2 hydrate for different granular composition. Fuel.

[53] Kim H.C., Bishnoi P.R., Heidemann R.A., Rizvi S.S.H. (1987). Kinetics of methane hydrate dissociation. Chem. Eng. Sci..

[54] Kutateladze S.S., Leont’ev A.I. (1989). Heat Transfer, Mass Transfer, and Friction in Turbulent Boundary Layers.

[55] Misyura S.Y. (2020). Comparing the dissociation kinetics of various gas hydrates during combustion: Assessment of key factors to improve combustion efficiency. Appl. Energy.

[56] Nyashina G.S., Kurgankina M.A., Strizhak P.A. (2018). Environmental, economic and energetic benefits of using coal and oil processing waste instead of coal to produce the same amount of energy. Energy Convers. Manag..

